# *Tuta absoluta* (Meyrick) (Lepidoptera: Gelechiidae) on the “Offensive” in Africa: Prospects for Integrated Management Initiatives

**DOI:** 10.3390/insects11110764

**Published:** 2020-11-06

**Authors:** Vimbai L. Tarusikirwa, Honest Machekano, Reyard Mutamiswa, Frank Chidawanyika, Casper Nyamukondiwa

**Affiliations:** 1 Department of Biological Sciences and Biotechnology, Botswana International University of Science and Technology, Palapye, Botswana; vimbai.tarusikirwa@studentmail.biust.ac.bw (V.L.T.); machekanoh@biust.ac.bw (H.M.); 2 Department of Zoology and Entomology, University of the Free State, P.O. Box 339, Bloemfontein 9300, South Africa; 2018066841@ufs4life.ac.za (R.M.); ChidawanyikaF@ufs.ac.za (F.C.)

**Keywords:** botanicals, invasive species, liabilities, pest management, tomato pinworm, natural substances

## Abstract

**Simple Summary:**

The past decade has seen Africa being invaded by an invasive and destructive insect pest of tomato, the South American tomato pinworm. To date, the pest insect has since spread to almost the entire continent at lightning speed. Farmers have responded to this pest pressure through the sole injudicious use of chemical pesticides. However, this method of control is expensive, not effective (owing to reported insecticide resistance) and has potential adverse effects on the environment (including humans). To mitigate this, more environmentally friendly, bio-based and sustainable alternatives need to be put in place. Natural substances (NSs), for example, the use of pesticidal plant extracts, naturally occurring antagonists and related substances, can be used in this regard. A literature review was conducted explaining various factors that contributed to successful invasion by the pinworm. The review also explored various control mechanisms (e.g., biological control agents) that can be used in combination with natural and other low risk substances in a holistic way for successful pest control. Focus was also taken on the enabling and limiting factors that influence farmers in embracing the use of these NSs in an integrated approach.

**Abstract:**

The South American tomato pinworm *Tuta absoluta* (Meyrick) has aggressively invaded the African continent. Since its first detection in North Africa in Morocco and Tunisia in 2008, it has successfully invaded the entire southern, eastern and western Africa, where it has been on the offensive, causing significant damage to Solanaceous food crops. While control of this prolific invader is primarily based on conventional synthetic pesticides, this form of control is consistently losing societal approval owing to (1) pesticide resistance development and consequential loss of field efficacy; (2) growing public health concerns; (3) environmental contamination and loss of biological diversity and its associated ecological services; and (4) unsustainable costs, particularly for resource-poor African farmers. As such, more ecologically sound pest management strategies, e.g., the use of natural substances (NSs), may offer a more sustainable approach to tackling this offensive. A systematic literature search through digital libraries and online databases (JSTOR, PubMed, Web of Science, SCOPUS and Google Scholar) was conducted using predetermined keywords on *T. absoluta*, e.g., South American tomato pinworm. We use this to explain the invasion of *T. absoluta* in Africa, citing mechanisms facilitating African invasion and exploring the potential of its control using diverse biological control agents, natural and low-risk substances. Specifically, we explore how botanicals, entomopathogens, semiochemicals, predators, parasitoids, host plant resistance, sterile insect technique and others have been spatially employed to control *T. absoluta* and discuss the potential of these control agents in African landscapes using more integrated approaches. We discuss the use of NSs as assets to general insect pest control, some potential associated liabilities and explain the potential use and barriers to adoption in African systems from a legislative, economic, ecological and social standpoint.

## 1. Introduction

Invasive insect pests are widely recognised as major threats to agricultural production, biodiversity conservation and the maintenance of ecological integrity [1,2,3]. Increased trade, human travel and globalisation have made crop production vulnerable to invasive alien insect pests [4,5,6]. In Africa, the rate of alien species invasion has dramatically increased in the past decades [7]. For example, “recent” invasive insect pest arrivals include *Chilo partellus* (Swinhoe) (Lepidoptera: Crambidae) [8], *Prostephanus truncatus* (Horn) (Coleoptera: Bostrichidae) [9], *Phenacoccus manihoti* Matile-Ferrero (Hemiptera: Pseudococcidae) [10], *Bactrocera dorsalis* (Hendel) (Diptera: Tephritidae) [11], *Spodoptera frugiperda* (JE Smith) (Lepidoptera: Noctuidae) [12] and indeed *Tuta absoluta* (Meyrick) (Lepidoptera: Gelechiidae) [13]. Given that ~70–80% of African livelihoods depend on agriculture [14], these invasions represent a significant biosecurity as well as food and nutrition security threat.

*Tuta absoluta*, commonly known as the South American tomato pinworm, is one of the most invasive destructive insect pests of tomato (*Solanum lycopersicum* (Solanaceae)) globally [13,15,16]. It is thought to have originated from the Andes region in Peru before spreading to Latin American countries during the 1960s [17,18]. Although it is an endemic Neotropical insect pest whose initial distribution was restricted to its native range in South America, it has successfully extended its geographical range following an unintentional introduction in the Mediterranean basin [15,19,20]. It acquired global pest status after detection in eastern Spain in late 2006 [15,21], following introduction as a single initial Chilean population in the early 2000s [22]. Thereafter, it rapidly spread throughout Europe [23,24]. Since then, the pest has rapidly spread east- and southward, tracking favourable biophysical environments [16]. During the last 10 years, *T. absoluta* has also spread in the Middle East and Asia at lightning speed, resulting in extensive naturalised populations in India, Iran, Israel, Syria, Turkey and Nepal [25,26,27,28,29]. Following its invasion in most European countries, *T. absoluta* successfully invaded the Afrotropics, reportedly via the Mediterranean Sea, with first detections reported in Tunisia, Algeria and Morocco in 2008–2009 [15,25,30,31]. Since then, further detections have been reported in Niger, Nigeria (2010), Senegal (2012) [32], Sudan, Ethiopia (2011) [32], Kenya (2013) [33], Tanzania (2014) [34], Uganda (2015) [33], Zambia, Botswana, Mozambique, South Africa, Malawi (2016) [13,35,36,37] and Angola (2017) [13], thereby elevating its continental pest status. This rapid north–south and downward incursion between 2008 and 2017 poses a biosecurity threat to natural and agroecosystems in pest-free countries. On a global scale, this pest is believed to have increased from primarily infesting only 3% of the worldwide tomato cultivated areas to 60% within 10 years [16], with the most recent in China, a major tomato growing country [38].

Underlying the invasion success of *T. absoluta* is its superior climate adaptation [39], polyphagous nature [40,41,42] and high biotic potential [43,44]. In response to *T. absoluta* infestations, chemical control is often used as the first line of defence [18,45], providing a quick fix to pest pressure. It is relatively easy to apply, readily available and may be cost effective when applied at a large scale. However, synthetic insecticides are expensive to many resource-constrained sub-Saharan African (SSA) farmers. The endophytic behavioural feeding and cryptic nature of *T. absoluta* larvae render the widely used contact insecticides ineffective [46,47,48]. Moreover, synthetic insecticides also affect (i) non-target biological diversity and their related ecosystem services, e.g., natural enemies and pollinators, thus disrupting desirable trophic interactions; (ii) development of pest resurgence; (iii) insecticide resistance development; and (iv) public health, owing to residual insecticide contamination [3,49,50,51,52]. Some of these vices have already been observed in *T. absoluta* chemical control, e.g., insecticide resistance [53,54] and non-target effects on beneficial fauna [55]. The practical implications of the European Union Directive 2009/128/EC on the sustainable use of synthetic pesticides and the future prospects that residue restrictions may become more stringent also necessitate the exploration of novel, sustainable non-chemical alternatives for the management of *T. absoluta* [56]. As such, efficacious, ecologically sound and safer biologically based or natural alternatives are required for sustainable management of *T. absoluta* for use in an integrated approach. Natural insecticides/substances (NSs) are thus compatible as they are cost-effective and eco-friendly owing to their rapid environmental degradation [57,58]. However, studies on the NSs that are effective on *T. absoluta* are scattered in literature, warranting an in-depth comprehensive review of the current methods. This study, therefore, provides a comprehensive, gleaned compendium of potentially effective NSs on this invasive quarantine insect pest. The understanding of the role and potential of NSs in limiting *T. absoluta* economic damage is important in establishing a baseline for sustainable management options. NSs widely used in pest management include microbial (e.g., entomopathogens and entomopathogenic nematodes), botanical (e.g., insecticidal plants or their derivatives) and semiochemical agents [59,60,61,62,63]. Despite availability of multiple potential options, current organic tomato production systems mostly rely on *Saccharopolyspora spinosa* (spinosad), *Azadirachta indica* (neem) and *Bacillus thuringiensis* toxins [16,64], signifying underutilisation of NSs. Moreover, identification and screening of effective locally available NSs remain scant in Africa [63,65,66]. Although NSs are environmentally friendly and have shown to be very effective in pest control, they have not been widely adopted by African farmers. Thus, this review also discusses *T. absoluta* invasion in Africa, possible mechanisms of invasion and the prospects of incorporating natural and low risk substances as assets in an integrated management approach. We also discuss barriers to the adoption and use of NSs in African agricultural systems from a legislative, economic, ecological and social standpoint.

## 2. Economic Impact of *Tuta absoluta* in Africa

Tomatoes are an important component of horticulture and a major pillar of sustainable development, with significant contribution to household and national food and nutritional security [42,67]. They are a cash crop grown for home consumption in the backyards of the majority homesteads across SSA and are an important source of vitamins [68]. Tomato production also significantly contributes to economic development ascribed to its high economic returns and ability to create employment (~60% of total labour force) and along the value chain [69,70]. About 170 million tonnes of tomatoes are produced worldwide [16,71]. Of this figure, Africa accounts for ~37.8 million tonnes annually. However, this figure is threatened by increased *T. absoluta* damage [29], with downstream consequences on African socio-economic value chains and household livelihoods [72].

*Tuta absoluta* larvae attack almost all aerial parts of tomato plants, resulting in ~80–100% yield losses if left uncontrolled [73]. Larval feeding also reduces fruit quality through creating pin holes prone to secondary attack by pathogens, rendering them unmarketable [74]. Increased cost of production has been reportedly experienced by small- and large-scale farmers in Africa due to increased costs for pesticides [62]. For example, recent evidence suggests decreased tomato yields and increased production costs [72]. Highest losses are mostly experienced during early invasion owing to inadequate mitigation measures related to lack of preparedness [18]. Nigeria experienced up to 80% losses in tomato produce in 2016 due to unfamiliarity with the pest and management strategies [70]. This reduced supply and catapulted an estimated 10-fold increase in tomato prices due to the laws of demand and supply [75]. Similarly, following *T. absoluta* invasion in South Africa (2016), pest-free countries banned importation of tomato and other Solanaceae crops from that country [13], resulting in significant economic losses. Similar losses have also been reported in Southwestern Angola [76]. Thus, *T. absoluta* pest pressure has negatively affected agricultural enterprises in Africa through direct losses and increased costs in pest management [29]. *Tuta absoluta* remains a pest of quarantine importance in countries under the Inter-African Phytosanitary Council (IAPSC) and has been reported on the European and Mediterranean Plant Protection Organization’s (EPPO’s) A2 list as a regulated insect pest [77].

## 3. *Tuta absoluta* Invasion Pathways and Distribution in Africa

Long-distance transmission and short-distance dispersal are the key drivers of *T. absoluta* invasion [78]. International agricultural trade is a key long-distance transmission mode that may have contributed to a larger extent in the introduction of *T. absoluta* into Africa, e.g., importation of fruits (e.g., tomatoes and egg plants) from pest-infested areas [29,79]. Other possible pathways for long-distance dissemination include production facilities and packaging materials (e.g., boxes, crates and pallets) from infested countries [43,80]. Hence, production facilities repack and distribute infested fruits, resulting in long-distance dissemination, reviewed in [81]. In addition, propagule material (e.g., seedlings), farm equipment and transportation vehicles from pest-infested areas are also possible pathways for long-distance transmission [78,79,82]. From the foregoing, the rapid spread across Africa may have been exacerbated by porous port of entries, weak phytosanitary regulations and ineffective early surveillance in the region [18]. Furthermore, natural factors (e.g., wind and water), larval crawling and adult flight are possible key short-distance dispersal pathways [78]. To date, *T. absoluta* has been reported in 41 of the 54 African countries (see [29]). The pest has not yet been officially reported in a few central and southwestern African countries [13,16]. However, cognisance of the widespread presence in Africa, this absence may be a consequence of lack of surveillance and pest detection mechanisms.

## 4. Factors Supporting *T. absoluta* Invasion in Africa

### 4.1. African Environments and Tuta absoluta Niche

**Figure 1:**
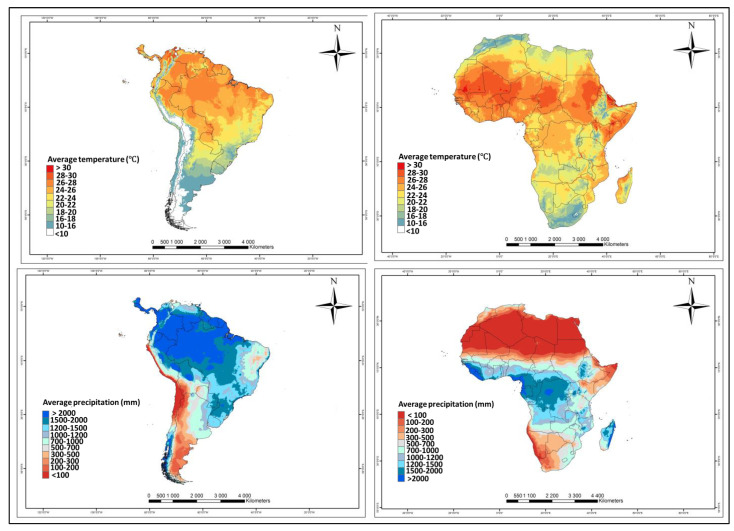
Temperature and precipitation comparison between the *T. absoluta* native (South America) and invaded (Africa) regions. The maps generally show that the *T. absoluta* niche is largely similar between the native and invaded region and thus successful establishment is possible. Maps were drawn in ArcGIS 10.3 from ESRI with data obtained from WorldClim Ver 2.0.

Interactions between the invader and the recipient agro-ecological regions influence pest establishment and spread, with the prevailing biophysical environment contributing to the invasion process [24,83,84]. Tropical regions, such as the majority of Africa, are highly vulnerable to insect pest invasions [24,85,86]. Among the key determinants of invasion success are climatic suitability, propagule pressure and the availability of suitable hosts [87,88]. Environmental matches between native and novel invasion areas play a pivotal role in invasion success [89]. Modelling studies have shown that temperature, relative humidity (RH) and hosts are critical determinants of *T. absoluta* successful invasion [24,43,90]. Research shows that Africa is a suitable biophysical niche for the pest [24,42,43]. Various models have been constructed to predict environmental suitability, potential and timing of *T. absoluta* spread in Africa [24,43,90]. Indeed, these models suggest temperature and RH environments for Africa are ideal for *T. absoluta* invasion and population establishment. However, it appears RH seems to have the strongest influence in *T. absoluta* invasion [24]. A comparison of temperature and precipitation between its native range and invaded African region shows a similarity in temperature ranges. However, the native range experiences higher precipitation as compared to the areas invaded in Africa (Figure 1). Nevertheless, reports suggest *T. absoluta* can thrive in high temperature and low precipitation environments as long as host plants are available [43]. The optimal temperature for its development is 30 °C, with egg, larval and pupal lower developmental thresholds at 6.9, 7.6 and 9.2 °C, respectively [91,92]. In addition, the upper developmental threshold from the egg to adult cycle is 37.3 °C [93]. Low temperature and high altitudes (>1000 m) are limiting factors for its survival, and a high RH is suitable for its development and life span [43,94,95]. The negative impact of rainfall on population abundance of *T. absoluta* has been reported [96]. Based on these ecological requirements and evidence presented in Machekano et al. [42], warm and humid conditions prevalent in Africa potentially elucidate its invasion success.

### 4.2. Physiological Tolerance

Environmental adaptations enable invasive species to overcome variable stressful barriers along the invasion continuum and significantly contribute to alien species propensity [97,98,99]. Overcoming different stressful environmental barriers is the first of several potential factors determining whether a species may become established, naturalised and ultimately invasive [83]. Temperature is the key abiotic “ecological filter” [100] for successful invasion in novel habitats [101]. As such, failure to mount any compensatory physiological resistance mechanisms against environmental barriers may offset invasion success [42,102]. Physiologically, some insect pests often increase invasion success through employing either increased phenotypic plasticity or basal stress tolerance [39,98,99,103]. A previous study has shown that *T. absoluta* larvae are more thermally plastic than adults and can shift their thermal tolerance in short and long timescales [39]. In addition, larvae showed a higher basal heat tolerance than adults, whereas adults recorded superior basal cold tolerance relative to larvae [42,104]. This physiological tolerance may help elucidate the notion that ecophysiology may have aided the current invasion by *T. absoluta*.

### 4.3. Increased Number of Generations

*Tuta absoluta* is an r-selected, multivoltine species [105] that remains active in Mediterranean and African winter climates [104,106,107]. Its pest status is largely associated with a high rate of reproduction, with each female producing up to 260 eggs during its lifetime [108]. Under the projected warming in Africa [109], this promotes an even shorter developmental time and higher voltinism. The life cycle of *T. absoluta* takes ~24–38 days at 27 °C, resulting in ~10 to 13 generations per year [15,16,91,106]. This rapid development may give *T. absoluta* a numerical advantage and propagule material that facilitates species proliferation.

### 4.4. New Niche with Limited Natural Enemies

**Figure 2:**
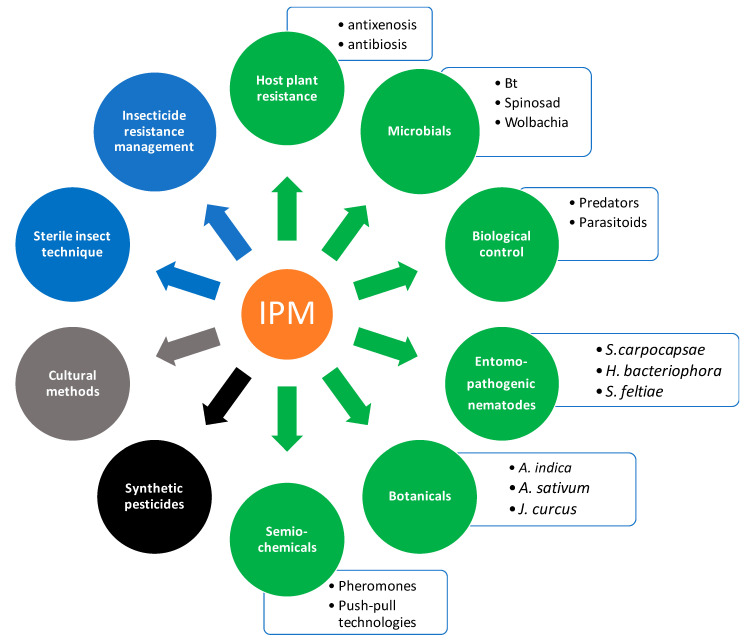
Schematic representation of proposed integrated pest management programme for *Tuta absoluta*. The emphasis is on the use of NSs, low-risk substances and biological control agents (green). Classic examples of “agents” or model organisms used within each category are given. A holistic use, in a judicious combination with target specific pesticides (black), environmentally friendly and low risk techniques (blue) and good crop management, (grey) may help in the IPM of this pest insect. These methods should be used in the presence of sound legislative measures that promote integrated approaches to pest management. Note, these examples are not purely exhaustive, but provide baseline information for IPM.

The enemy release hypothesis postulates that invasive species likely have reduced biotic pressure (e.g., natural enemies) than their native counterparts [110]. It also follows that parasitoids and predators (see [111,112]) specific to the invasive species may be absent in the novel areas, resulting in the pest not being suppressed [113] owing to a lack of co-evolved biological control agents following invasion [114]. This is likely the reason behind the limited natural biotic pressure on *T. absoluta* in Africa, consequently contributing to its quick establishment [72]. The enemy-free hypothesis also states that invading species often perform better and experience rapid population growth in new areas [115,116,117]. This is because in the new habitats, invading species may at most encounter opportunistic generalist natural enemies in their new range, but not more efficacious coevolved specialists [113,116,117,118]. Indeed, various indigenous generalist predators and parasitoids have been recorded as potential biological agents of *T. absoluta* in some parts of Africa, albeit with low efficacy [13]. However, these studies remain scant and constrained in space [72]. Various authors have reviewed the natural enemy complex of *T. absoluta* [15,16,119,120]. Amongst these species, a few have been recorded in Africa. The few documented in the continent are mostly prevalent in North Africa [13]. The generalist mirid predator *Nesidiocoris tenuis* (Reuter) (Hemiptera: Miridae) has been the most reported, albeit also confined to North Africa [13,121]. Other predators recorded include *Macrolophus pygmaeus*, (Rambur) (Hemiptera: Miridae), *Macrolophus caliginosus* (Wagner) (Heteroptera: Miridae), *Dicyphus tamaninii* (Wagner) (Heteroptera: Miridae), *Rhynocoris segmentarius* (Germar) (Hemiptera: Reduvidae) and *Dicyphus errans* (Wolff) (Hemiptera: Miridae) [13]. In Africa, the parasitoid complex that has so far been recorded through field monitoring and surveys include larval braconid parasitoids of the genus *Apanteles* and *Bracon* in Nigeria; egg parasitoids of the genus *Trichogramma* in Nigeria, Tunisia, Algeria and Morocco [13,122]; and several hymenopterous parasitoid species belonging to different families (Kenya), including *B. nigricans*, *B. hebetor*, *Dolichogenidea appellatory* (Telenga) (Hymenoptera: Braconidae), *Ecdamua cadenat* (Risbec) (Hymenoptera: Torymidae) and *Neochrysocharis formosa* (Westwood) (Hymenoptera: Eulophidae) in Sudan [13]. However, reports suggest these parasitoids cannot solely provide effective *T. absoluta* control [13]. Thus, use of other complementary NSs combined in an integrated approach may improve efficacy and sustainability in *T. absoluta* management (Figure 2). To our knowledge, no egg parasitoids have been recorded in southern Africa. Thus, the inefficiency by current spatially isolated indigenous natural enemies coupled with total absence in some regions [13] has provided an enemy-free platform for *T. absoluta* successful invasion and establishment.

### 4.5. Wide Host Range

*Tuta absoluta* is polyphagous, exploiting a wide range of alternate hosts [123,124]. This behaviour allows the continuous and spatial omnipresence of the pest. Although Özgökçe et al. [41] reported 26 different host plant species for *T. absoluta*, it has a strong preference for solanaceous species, with tomato, potato (*Solanum tuberosum*) and European black nightshade (*Solanum nigrum*) being the most preferred [15,125]. In addition, it can also oviposit and develop on several plants belonging to the Amaranthaceae, Convolvulaceae, Fabaceae and Malvaceae [16]. Since *T. absoluta* is rapidly and continually evolving, evidence suggest it is also expanding its host range [124]. For example, in Sudan, *T. absoluta* was recorded on watermelon (*Citrullus lanatus*) and alfalfa (*Medigo sativa*) [40] and on weeds such as thorn apple *Datura stramonium* [124]. In Algeria, Drouai et al. [126] found *T. absoluta* on beet (*Beta vulgaris*), spinach (*Spinacia oleracea*) as well as the weed species *Chenopodium bonushenricus* and *C. rubrum*. In South Sudan up to 50% of the *T. absoluta* damage was observed on potato foliage [30]. In Botswana, *T. absoluta* was spotted on some wild hosts, *Solanum aculeatissimum* (Jacq.), *Solanum coccineum* (Jacq.) and *Solanum supinum* [42]. Apart from the main solanaceous plant hosts, *T. absoluta* affects other crops of economic importance (Table 1). The availability of alternative host plants is an important factor that allows the sustainability of the pest in the absence of the primary tomato host [42,127]. Therefore, the presence of a wide range of both cultivated and wild host plants in African landscapes creates a refuge opportunity for host switching and consequent pest success.

### 4.6. Pesticide Resistance

Synthetic insecticides are employed as the primary method of control against insect pest infestation [2,137]. For example, Tunisia registered 18 new insecticides during 2009–2011 following *T. absoluta* invasion although they all turned out ineffective [25,138]. Intensive use of synthetic insecticides for *T. absoluta* management coupled with insect biological traits, such as a high reproductive potential and multivoltinism, endophytic larval feeding behaviour and mining habit as well as polyphagy, has increased *T. absoluta* selection pressure for insecticide resistance [53,139,140]. In South America and Europe, resistance has been reported against conventional insecticides such as organophosphates (OPs), pyrethroids, cartap, diamides and avermectins [16,46,139,141,142]. The main resistance mechanisms evolved through altered target-site sensitivity and/or enhanced detoxification, depending on the chemical class [64]. In northern Nigeria, resistance was reported in cyhalothrin (a Type II pyrethroid), propoxur and chlorpyrifos-methyl via enzyme mutation, underlying the challenges in managing this invasive pest using pesticides [70]. Given the prohibitive costs of synthetic pesticides for African farmers, evolution of pesticide resistance will further compound losses on already resource-constrained farmers.

## 5. Potential Use of Natural Substances

### 5.1. Botanicals

Botanical insecticides are naturally occurring chemicals extracted from plants with insecticidal properties [61,143]. They can be classified based on their chemical constituents into categories, namely, essential oils, flavonoids, alkaloids, glycosides, esters and fatty acids [144,145,146,147,148]. Plant derivatives and bioactive compounds have been used to manage different crop pests with notable success [63]. Their physiological effects on insects vary depending on the target site and mode of action [61], with most acting as repellents, feeding deterrents/antifeedants, toxicants, growth retardants, chemosterilants and attractants [61,63,149]. Plant parts used are dependent on the targeted bioactive compounds as well as their localised concentrations. However, use of barks, leaves, roots, flowers, seeds and stems is widely reported [63]. Common botanicals with reported insecticidal properties include neem (*A. indica*), garlic (*Allium sativum*), ginger (*Zingiber officinale*) and pyrethrum (*Tanacetum cinerariifolium*) [63,143].

Given the wide availability of botanicals, ease of application and low operational costs, botanicals are a viable option for sustainable *T. absoluta* management. *Azadirachta indica* and *Jatropha curcas* extracts have been reported as efficacious on *T. absoluta* eggs and larvae [150]. In an empirical study, four-day exposure to *J. caurcas* and *A. indica* seed extracts resulted in 18% and 25% egg mortalities, respectively, whilst 24-h treatment elicited larval mortalities ranging from 23.5 to 48.5% and 33 to 46.7%, respectively. In another study, Abdel-baky and Al-Soqueer [151] showed that simmondsin extracts, obtained from seeds of Jojoba, *Simmondsia chinensis* L., were effective in controlling 2nd instar larvae of *T. absoluta*. Field studies showed 71.82 and 74.26% larval mortalities following treatment using the biopesticides *A. sativum* and *A. indica*, respectively [152]. Furthermore, ethanolic leaf extracts of *Piper amalago* var. *medium*, *P. glabratum* and *P. mikanianum* significantly elicited *T. absoluta* larval mortalities [153]. *Piper* species have amides (e.g., piperamides) that are known to have neurotoxic and lipid metabolism effects [154], manifesting as knockdown and paralysis followed by death [155]. Several other plant extracts have been found to be effective against *T. absoluta* (Table 2). Given the efficacy of some of the reported botanicals, e.g., [151] (Table 2), using them in combination with soft pesticides and in an integrated approach could provide more optimised control. However, despite empirical support for the botanicals’ efficacy, their use in agriculture is currently limited in commercial use on vegetable and fruit crops with few prospects for commercial development of new products [143]. Several factors affect the wide success of botanicals as conventional pesticides; for example, availability of plant material, solvent types, rapid environmental degradation, registration bureaucracy, market opportunities and availability of competing products are some of the barriers to successful use of botanical insecticides [143].

### 5.2. Entomopathogens

Entomopathogens, such as *Metarhizium anisopliae* var. *anisopliae* (Metsch.) Soroki, *Beauveria bassiana* (Balsamo) Vuillemin and *Bacillus thuringiensis* (Berliner) have shown efficacy against *T. absoluta* (Table 3) [159,160]. The soil dwelling bacterium *B. thuringiensis* is one of the most important microorganisms with entomopathogenic properties [161]. It is environmentally friendly and can be extensively used as part of an integrated approach to pest management [81]. Apart from Lepidoptera, *B. thuringiensis* was reported to exhibit insecticidal effects on many other insect orders [161], making it ideal for sustainable integrated management. Indeed, both *B. bassiana* and *B. thuringiensis* have been used to control an array of pest insects, including whiteflies, thrips and termites [162,163]. *Bacillus thuringiensis* has already been widely used in *T. absoluta* control [160,161]. Efficacy studies of *B. bassiana* and *B. thuringiensis* on *T. absoluta* showed that third instar larvae were most susceptible [60]. Furthermore, their interaction effects were synergistic in *T. absoluta* control. By contrast, Gonzalez-Cabrera et al. [160] found evidence that first instar larvae were the most susceptible to *B. thuringiensis* and that it could keep *T. absoluta* below economic thresholds [164]. Similarly, Biondi et al. [16], reported that *Wolbachia* bacterial infection may potentially be efficacious for *T. absoluta* through its effects on reproduction. Spinosad, a fermentation product of *S. spinosa*, has also been used in *T. absoluta* control [165,166,167]. However, its continued use has been threatened by resistance [168]. Thus, there is need for complementary control options to fight *T. absoluta*, if pest populations are to be maintained below economic threshold levels [16]. Regardless of sporadic resistance reports for these substances, they still remain reliable and efficacious sustainable options to pest control in integrated systems.

### 5.3. Entomopathogenic Nematodes (EPNs)

Entomopathogenic nematodes (EPNs) (Table 3) are biological control agents that can kill insect pests using their coevolved mutualistic intestinal bacterium [174,175,176]. Their use in pest management is already widespread and have shown efficacy in diverse taxa [177]. Entomopathogenic nematodes have been used against similar Lepidopterans, e.g., false codling moth (*Thaumatotibia leucotreta*), codling moth (*Cydia pomonella*) and the sugarcane borer (*Eldana saccharina*) [176,178,179]. Recent studies have shown the EPNs *Steinernema feltiae*, *S. carpocapsae* and *Heterorhabditis bacteriophora* are effective against all larval instars of *T. absoluta* (Table 3) [177]. Similarly, Kamali et al. [62] reported high efficacy for *S*. *carpocapsae* and *H. bacteriophora* against fourth instar larvae. These results suggest scope for EPNs in *T. absoluta* management and can be an intergap component of an integrated management approach.

### 5.4. Semiochemicals

Semiochemicals are chemicals mediating interactions across organisms by eliciting behavioural responses in recipient organisms within and across species. Sex pheromones have been, by far, the most widely used semiochemicals in pest management [59]. Semiochemical-based management of insects usually include pheromone lure and kill, mass trapping and disruption of mating activities [47,180]. Pheromones can also be effectively utilised in population monitoring to determine action thresholds, early pest detection and other manipulations of insect pest behaviour [181]. This form of control has been successfully implemented in South America, Europe, Asia and Africa in managing *T. absoluta* in greenhouses and open fields [42,81]. Using sex pheromones, Filho et al. [182] recorded 233 males/trap/day under greenhouse conditions. These trap catches can be used as part of a mass trapping *T. absoluta* control strategy. Pheromone-mediated control of *T. absoluta* is more recommended as a supplementary measure in combination with other management options. For example, a study in Egypt showed that a combined use of sex pheromone and other insecticides is effective against *T. absoluta* larvae [183]. Similarly, Cherif et al. [184] showed that mass trapping in combination with *B. thuringiensis* and cyromazin significantly reduced *T. absoluta* numbers. Other behaviour-modifying strategies, e.g., the push–pull strategies, have worked successfully for the management of other Lepidopteran pest insects [185]. However, to our knowledge, empirical studies showing the efficacy of a push–pull strategy in *T. absoluta* control in Africa is missing. Moreover, the use of a pheromone-mediated management system in Africa is also low due to high costs and limited availability. Nevertheless, semiochemicals represent an efficacious and sustainable approach to pest management.

### 5.5. Sterile Insect Technique (SIT)

Sterile insect technique is an environmentally friendly control option aiming to suppress pest populations through F_1_ generation sterility enabled by release of sterile males that mate with wild females [186], thereby producing non-viable offspring [186,187,188]. However, male sterilisation can reduce fitness thereby compromising field competitiveness [189]. As such, various mechanisms have been put in place to compensate for this reduced fitness and maintain optimal SIT efficacy in the field, including the use of low dosage gamma radiation and thermal preconditioning/acclimation [186,190].

Sterile insect technique has been used successfully for control of Lepidopterans, e.g., pink bollworm, *Pectinophora gossypiella* (Saunders) (Lepidoptera: Gelechiidae) [191]; false codling moth, *T. leucotreta* (Lepidoptera: Tortricidae) [192]; and codling moth, *C. pomonella* (Lepidoptera: Tortricidae) [193]. The technique can be used in an area-wide management approach, incorporating other options such as natural enemies, cultural control and the application of bio-rational pesticides [194,195]. Cagnotti et al. [191] reported that the inherited sterility control of *T. absoluta* can be combined with the use of the predator *Tupiocoris cucurbitaceus* in pest management. Given the efficacy of SIT in pest management, its environmental soundness coupled with a high compatibility with other control measures, SIT warrants exploration for use in *T. absoluta* management in Africa.

### 5.6. Host Plant Resistance

Development of tomato cultivars resistant to pests is an important strategy in pest control using NSs and is one of the fundamental pillars of an integrated management approach [196]. While few moderately resistant tomato cultivars have been reported [197,198], the majority are highly susceptible to *T. absoluta* infestation. Three mechanisms may account for plant resistance to insect attack, namely antixenosis, antibiosis and tolerance [199]. Thus, tomato cultivars significantly differ in their susceptibility to *T. absoluta* [200,201,202,203], and that resistance is positively associated with trichomes density and the diversity and concentrations of host secondary metabolites [201,202,204]. Indeed, tomato plants possess glandular trichomes that produce volatile and non-volatile secondary metabolites, e.g., acyl sugars, terpenoids, phenylpropanoids, flavonoids and phenolic compounds [200,205]. Therefore, breeding programmes have targeted lines with high acyl sugars and other secondary metabolites, e.g., zingiberene for host resistance against pests in tomato production [16,136]. Use of resistant varieties may also offer a cost effective and sustainable approach to the fight against the *T. absoluta* scourge in Africa.

### 5.7. Use of Predators and Parasitoids

Several predators and parasitoid biological control agents have been demonstrated to suppress populations of the tomato pinworm below economic threshold levels (Table 4). Conservation and augmentative biological control programmes using predators and parasitoids have been developed for *T. absoluta* following its invasion in Europe [16] and Africa [31,119,206,207]. Hemipteran predators, notably anthocorids, geocorids, mirids, nabids and pentatomids, have been identified as *T. absoluta* biocontrol agents in both native and invaded areas [15,16]. The generalist predators *M. pygmaeus*, *N. tenuis* and *Dicyphus* spp. [208] are the most common antagonists in European greenhouses [119]. Their use has been complemented with *B. thuringiensis* sprays against the early larval infestations [209]. Adults and nymphs of *M. pygmaeus* and *N. tenuis* prey on *T. absoluta* eggs and larvae (preferably first instar) [206,210]. *Dicyphus tamaninii* has been reported as a predator of *T. absoluta* eggs and larvae in North Africa [206]. Other predators reported in Africa include *N. tenuis*, *M. pygmaeus*, *M. caliginosus*, *D. tamaninii*, *R. segmentarius* and *D. errans* [13]. In Spain, predatory lacewings (*Chrysoperla carnea*) and mites (*Amblyseius swirskii* Athias-Henriot and *A. cucumeris* Oudemans) have been reported as egg and larval biocontrol agents. Furthermore, predatory ants, e.g., *Tapinoma nigerrimum* (Nylander) has been identified as a biocontrol agent in North Africa (Table 4). Where available, conservation of these predators as NSs or mass rearing for use as augmentation or inoculative releases may offer a sustainable approach to the management of *T. absoluta*.

Hymenopteran egg parasitoids belonging to the genus *Trichogramma* have been reported as efficacious biological control agents in protected tomato crops [211,212]. For example, *Trichogramma pretiosum* (Riley) and *Trichogramma achaeae* (Nagaraja and Nagarkatti) have been used in *T. absoluta* management in both native and invaded European regions [213,214]. In Africa, egg parasitoids comprising especially *Trichogramma* spp. have shown potential for mass releases, e.g., in Tunisia [215]. Indeed, reports suggest that the parasitoid complex for larval *T. absoluta* comprise approximately 20 hymenopteran species (Table 4) [207,216,217]. Similarly, several *T. absoluta* larval parasitoids, including Eulophids, Braconids and Ichneumonids, have also been reported in the Mediterranean basin [206]. For example, the idiobiont *Necremnus artynes* parasitising third instars is widely documented [218]. Similarly, in Egypt, the larval parasitoid *Stenomesius japonicus* has been reported a desirable NS for *T. absoluta* biocontrol [206], while *N. formosa* (Westwood) has been reported in the Palearctic area, Asia, Africa and North America [119]. As part of classical biocontrol, the larval parasitoid *Dolichogenidea gelechiidivoris* Marsh. (Syn.: *Apanteles gelechiidivoris* Marsh) was introduced from Peru into Africa [72]. Progressive work has shown that *D. gelechiidivoris* prefers first and second instar larvae and is a highly efficacious parasitoid [72]. Conversely, few *T. absoluta* pupal parasitoids have been reported [16]. Current research suggests Braconids, Chalcidids, Eulophids and Ichneumonids as potential pupal parasitoids [15,16,119,219] (see Table 4). Given the diversity of African landscapes, and the diversity of both indigenous and exotic natural enemies as potent as NSs in pest control, using these in combination with other compatible NSs may be key to integrated management of *T. absoluta* in Africa.

## 6. Use of Synthetic Pesticides and Integrated Pest Management

Regardless of their limitations, chemical pesticides continue to be an important component of integrated pest management (IPM) and can be a crucial pillar in *T. absoluta* management. Common active ingredients registered for *T. absoluta* control include pyrethroids [51], organophosphates [3] and diamides [54]. Laboratory bioassays using insecticides with some of these active ingredients showed very high efficacy for *T. absoluta* control [29,70,234,235,236]. Despite high laboratory efficacy, field *T. absoluta* optimal control remains a challenge owing to their cryptic leaf mining behaviour that renders contact insecticides ineffective. In addition, continuous use of these pesticides has also led to resistance development. Insecticides may also have adverse effects on the environment, beneficial arthropods and public health [237]. Thus, minimal insecticidal use is recommended in an IPM approach, which should rather be complemented with more environmentally benign NSs.

IPM encourages the use of eco-friendly strategies, such as NSs in biological control, used in compatible combinations with other efficacious methods, including the use of selective insecticides for pest control [238]. Thus, IPM comprises a cocktail of control practices, which may include cultural, chemical and biological control for the management of an economic pest species (Figure 2) [239]. For *T. absoluta* management in Africa, and cognisant of all available control options discussed above, we propose the combination of compatible methods in an IPM approach and incorporating the use of sustainable NSs (see Figure 2). This proposition is environmentally friendly, conserves biological diversity, including natural enemy populations, and presents little potentially negative public health implications. With the demerits associated with synthetic insecticides (see, e.g., [53,164]), IPM approaches may provide more lasting sustainable solutions to increased *T. absoluta* pest challenges [15,60] and indeed other pest species.

## 7. Potential for Natural Substances in Pest Control: Assets and Liabilities

### 7.1. Legislative and Regulatory Frameworks

Despite being major pillars to an integrated approach to *T. absoluta* sustainable management, there are major liabilities and bottlenecks associated with NS development and successful deployment in Africa. Some major liabilities for NSs include the increased number of complex guidelines, regulations and inadequate lobbying by biocontrol champions [240,241,242,243]. Furthermore, negative and often conflicting effects on chemical industry profitability and general farmer overreliance on pesticides [137,242,244] are some of the barriers to NSs, biological and low-risk pest management options. Policy guidelines in the production, exportation or importation, shipment, environmental risk assessment and field application of NSs are bureaucratic [240,242,243,244]. Except for Kenya [245], policy and legislation governing NSs in Africa are mostly based on templates for synthetic chemical pesticides with a single active ingredient, and thus does not permit registration of NSs with complex multiple active compounds [245,246]. This impedes the ability of resource-poor small and medium enterprises (SMEs) active in the NSs crop protection industry in African countries to register their products [241,246]. Governments need to create enabling legal and policy frameworks as pre-requisite for the promotion of the NS industry, particularly promoting existing SMEs [247] relative to multi-national companies. Available evidence suggests that the multi-national companies in the agrochemical industry are “unwilling” participants in creating an enabling legislative and registration environment for the NSs motion [246]. Benjamin and Wesseler [244] suggest that slow adoption of NSs may be caused by prolonged and prohibitive regulations. Therefore, changes in regulatory policies governing NSs in SSA should aim to reduce this bottleneck and make the toxicological evaluation regulations light touch [240,241,245,246]. In light of this, African governments remain the main change agents [242,244,247] through policy and regulatory adjustments that promote production, registration and marketing of NSs [246,247].

In some cases, policies on NSs and IPM regulatory guidelines are elaborate on paper but lack implementation [246,247,248]. This, in part, is supported by the lack of regulation on importation, trade and use as well as residue monitoring of unregistered/highly toxic pesticides [45,246]. This may be largely due to a lack of funding [242,247]. Therefore, monetary policies should enact direct funding of implementation of the proven NSs packages. Increased national funding will enhance research and development on NSs and their derivatives [246]. However, research and developments funds are still limited in SSA due to restrictions attached to donor funds [246,249].

Bureaucracy, inefficiency and prolonged government legislative processes are additional impediments to swift changes in NSs policies and legislative regulations [242,246,250]. In addition, difficulty in harmonising regional polices due to fundamental mismatches or disagreements inhibits efficient movement of NSs across borders for purposes of research, propagation, pilot tests or field applications [246]. Sub-Saharan Africa member states may need to adopt a common approach in interpretation of the Acts governing NSs, e.g., harmonised data requirements in toxicological studies or adoption of standard application forms and dossier formats for importation of NSs [240,241,243]. In addition, establishment of a central panel of pan-SSA experts may need to be developed to overlook NSs submissions [243]. If data requirements are harmonised, NSs approved in one country may be accepted in another without the drudgery and costs of repeated efficacy evaluations [241,243].

Encouragingly, related policy structural guidelines are in place for some countries [242]. For example, the organic production of fruits and vegetables, e.g., in Kenya [245,246], has very strict existing legislative measures that may be utilised as pedestals (asset) for building new guidelines for policy adjustments for NSs research and development [241,242,247,249]. Existing policies on conservation of biodiversity and protection of endangered species may be fundamental springboards for policy adjustments for the promotion of NSs. In addition, the Food and Agriculture Organisation (FAO) and Word Health Organisation (WHO) guidelines are freely available for adoption and modification where necessary [251]. The public outcry against pesticide-contaminated food and environment degradation reviewed in [252,253] may also be used as bottom-up pressure to justify policy modifications, promoting the use of NSs. For example, SSA governments can use the substitution principle, by replacing the most toxic pesticides (e.g., WHO hazard classes I and II) with the promising NSs [242,254]. Alternatively, governments can increase tax on the most toxic pesticides and use that revenue to provide subsidies and financial incentives to early adopters or pesticide companies involved in the development of NSs [242,254,255]. As the development and uptake of efficacious NSs increase, this may also positively affect farmer behaviour towards shifting from conventional pesticides to adoption of NSs.

Limited adoption in use of authorised pesticides of NSs is partly attributable to a lack of education and information [45,256,257,258]. However, evidence exists that some farmers in SSA are traditionally using NSs without anecdotal efficacy and safety validations [246,247,248,249,250,251,252,253,254,255,256,257,258,259]. Based on this, SSA governments and other stakeholders alike need to develop and consolidate NS-oriented educational programs to increase ecological literacy and reduce adoption barriers [242,256,258]. It is therefore in the interest of SSA countries to modify policies enabling the development of simplified testing and validation protocols to legitimise existing NSs for improved use and expansion for the greater good.

### 7.2. Economic Dynamics

The economic feasibility of NSs is ingrained in a functional biopesticide industry that, in turn, is rooted in SMEs and small-scale farmers or farmer groups [247,249,260]. This is because large companies are sceptical about the return on investment in NSs due to uncertainties regarding the market size (adoption issues), consistent supply of raw materials, uncertain patent issues and less than absolute efficacy [242,247,259]. SMEs in SSA are not well developed due to financial constraints against the backdrop of prohibitive costs of investment on research and development for NSs [240,247,249]. Consequently, instead of local production, most SSA agro-companies survive on distributing internationally sourced products [247,259], missing huge local investments opportunities. For example, *B. thuringiensis* (FlorBac WG^®^) and Spinosad (*S spinosa*, Spintordust^®^) distributed by SSA subsidiary agrochemical companies are produced outside the continent [246,247]. This partly contributes to their premium prices, likely aiding their economic non-viability for the majority of resource-constrained farmers in SSA.

Estimation of the full economic value of NSs as a pest management tool is complex and depends on several factors, including the product being protected, the enterprise (e.g., farm), community structure to societal costs and benefits, as reviewed in [244,248]. However, empirical evidence exists in support of financial sustainability for NSs versus conventional insecticides. For example, Amoabeng et al. [249] showed that pesticidal plants were more cost effective than synthetic insecticide in the control of the pests *Brevicoryne brassicae* (L.) (Hemiptera: Aphididae) and *Plutella xylostella* (L.) (Lepidoptera: Plutellidae) in cabbage. Similarly, McConnachie et al. [261] showed that the net present value of using a biocontrol agent *Stenopelmus rufinasus* (Gyllenhal; Coleoptera: Curculionidae) against the red waterfern *Azolla filiculoides* was US$1093 per hectare and US$206 million for the entire South Africa (with a benefit–cost ratio ranging from 2.5:1 to 15:1). While this study documented noble cost savings, Naranjo et al. [248] argues that the economic benefits of NSs are even higher. For example, by factoring avoided costs, e.g., public health protection, environmental damage costs, other maximum incremental social tolerable irreversible costs (MISTICs) and maintenance of essential ecological services [244,262]. Factoring in all these variables thus reflects NSs may be way cheaper both in the short [249] and long term [242,244,263] than conventional pesticides. These arguments reinforce the overall economic viability of NSs as a critical pillar for IPM [242,244,261].

The market for NSs in SSA is underdeveloped, small and highly fragmented due to lack of investment, low ecological consciousness and farmers’ skewed behavioural perceptions towards pesticides preference, e.g., [246,249,255], relative to NSs [264,265]. For example, in Ghana, only 14–25% of the farmer population are using NSs [249]. While studies show that this value may increase with increased education on NSs [242,244,246,249,259], limited funding and stringent regulatory processes still remain significant bottlenecks [244,246,259]. We thus suggest the use of a combination of enabling policy adjustments and direct funding and incentives for biocontrol practitioners to improve NS adoption. Through incentivising SMEs and mobilising farmer or community groups, SSA governments can encourage NS enterprises [249]. This system will build a strong localised production and trading system that can work as an encouraging baseline for private sector investment for scaling up [246,248]. Previous studies suggest scaling up is the main pitfall for research and development initiatives on NSs in SSA [259]. Thus, this can be circumvented by managing the production and trading of NSs using economic product development pathways and commercial imperatives wrapped around government-supported SMEs.

### 7.3. Ecological Perspectives

The main ecological assets of NSs in SSA include existing freely available rich biodiversity, known trophic systems and established harvesting and rearing/propagation protocols [246,247,250]. Most NSs are highly specific, e.g., EPNs [264], microbial pesticides and parasitoids [265,266]. Thus, they have low impact on beneficial and non-target species [252,266,267,268] and hence contribute to maintenance of environmental integrity and conservation of biological diversity [242,257]. In addition, production of NSs is ecologically benign and has a low carbon footprint [267]. Plant-based NSs, e.g., natural enemies, have the ability to sustainably self-propagate and disperse in space through inoculative releases [247,250]. This makes NSs a cost-effective management tool. Exhaustive benefits and assets of various NSs for the developing countries have fully been documented [241,248,252,266,269].

The liabilities of some NSs, e.g., predators and parasitoids (Table 2, Table 3 and Table 4), include dependency on the host pest insect for survival [264,265,270]. This therefore means NSs need the presence of the pest, mediated by density-dependent factors and thus allowing some level of tolerable pest damage. Thus, use of preventive measures for pest management are not possible when using NSs. The action of NSs may also be slow when compared to synthetic insecticides, allowing some degree of pest damage on products. This may be undesirable for pest outbreaks that require rapid efficacy to bring populations below economic thresholds. Furthermore, this may also offset the quality of cosmetic products, e.g., the fruit and vegetable industry, whose Economic Injury Levels (EILs) are low [246]. In addition, some NSs like parasitoids, EPNs and baculoviruses are highly sensitive to the biophysical environment [264,265,266,271], and thus their efficacy may vary in space. This makes the deployment of NSs challenging in largely rainfed, dry cropping systems in SSA [246] or under rapidly shifting climate environments. Similar environmental limitations for NSs have been made for the biocontrol agents *Romanomermis culicivorax* [265], *Steinernema carpocapsae* [272] and other biocontrol approaches [271,273,274,275].

Other ecological limitations of NSs include a lack of sound and sustainable mass rearing and/propagation technologies that facilitate reliable product supply and reduce the costs of importation while safeguarding ecosystems [246,247,250]. Use of botanicals as natural pesticides has been criticised owing to unsustainable harvesting of bioactive plant species [246,249,250]. In some cases, effective pesticidal plants that are not native to production areas, e.g., in a push–pull system [185], may become introduced weeds, thus increasing the cost of production. Lack of expertise and experience in the phytochemistry of plant-based NSs for extraction of active ingredients has also been a barrier to success. This expertise is needed for robust evidence-based safety screening to improve research and development processes.

### 7.4. Farmer Perceptions and Social Dynamics

Natural substances are readily available for most small-scale, resource-poor farmers, and thus argued to be the most appropriate technology compatible with SSA [247]. The biggest asset for NSs is its relatively low public health risk. Small-scale farmers are largely poorly advised on the appropriate use and disposal of toxic synthetic pesticides [45,137,276]. Compounded by the aggressive promotion of synthetic pesticides that overshadows NSs [244,246,259,277], this makes the use of pesticides a significant public health threat for livelihoods in SSA. There is also limited infrastructure bridging researchers and farmers to facilitate knowledge transfer. As such, most research outputs on NSs remain as pilot projects or scholarly papers that are inaccessible to the small-scale farmers [244,257,259]. Nevertheless, behavioural studies show that while SSA farmers are solely used to pesticides, they are willing to adopt NSs if proven efficacious [45,137,276,278]. Players in research and development thus need to lobby for uptake of newly developed NSs technologies through engagement with relevant stakeholders.

Fluctuations in supply in NSs, e.g., botanicals, often reduce reliability of this tool for pest management [265]. Furthermore, vulnerability to the biophysical environment often compromises efficacy [248,265], leading to poor product quality and low market values [249]. The application of some NSs also requires significant adjustments in current farming methods, labour and skills [277]. For example, the use of parasitoids requires shift from monoculture to habitat management that encourages biodiversity conservation [265,279]. Without change in perception/behaviour, ecological literacy and financial incentives, farmers may not be socially ready for the changes facilitating the use of NSs. Farmers’ beliefs, attitude and behaviour are major factors influencing adoption of NSs. Creative persuasion of the non-conformers to NSs is needed to change their choice of pest management approaches [242,244,277]. Indeed, Goldberger et al. [277] showed that out of the three farmer categories, the “environmental stewards” and “networking farmers” are likely to partake in NSs compared to those farmers solely looking to maximise production. Similarly, mobilising farmers into networking groups may increase NS adoption [256,277], e.g., establishing social/networking groups that can implement specific NSs may be a fruitful endeavour for SSA [255,256]. These groups also facilitate ease of training and awareness campaigns on ecological literacy, economic feasibility and other assets of NSs.

## 8. Conclusions

The continental invasion of *T. absoluta* represents a significant biosecurity threat that affects the majority of livelihoods dependant on agricultural sustenance. A number of biological and physiological factors discussed here may, in part, contribute to its current spread in African natural and agro-ecosystems. Insecticide use against *T. absoluta* has been the common default response to pest pressure. However, pesticide misuse affects the environment, public health, ecosystem services and often leads to pest resistance development. Thus, use of low-risk NSs and biocontrol methods in an integrated approach may be the sustainable solution to the *T. absoluta* problem in SSA. However, NSs also have their own liabilities as a pest management tool in African systems, argued from a legislative, economic, ecological and social standpoint. Elucidating these factors is critical in facilitating research and development of NSs in Africa and their consequent adoption as a sustainable tool for pest management.

## Figures and Tables

**Table 1:** Alternative host plants of *Tuta absoluta* reported in the literature. The list may not be purely exhaustive but was compiled using the literature available at the time of writing.

Family	Host Plant	Reference
Solanaceae	*Solanum tuberosum* L.	[15]
	*Solanum nigrum* L.	[125]
	*Solanum melongena* L.	[128]
	*Solanum aethiopicum* L.	[96]
	*Solanum anguivi* Lam.	[82]
	*Solanum macrocarpon* L.	[82]
	*Solanum scabrum* Mill.	[82]
	*Solanum villosum* Mill.	[82]
	*Solanum aculeatissimum* (Jacq.)	[42]
	*Solanum coccineum* (Jacq.)	[42]
	*Solanum supinum* Dunal	[42]
	*Solanum americanum* Mill.	[128]
	*Solanum bonariense* L.	[129]
	*Solanum elaeagnifolium* Cav.	[129]
	*Solanum gracilius* Herter	[129]
	*Solanum hirtum* Vahl	[130]
	*Solanum pseudo-capsicum* L.	[129]
	*Solanum sisymbrifolium* Lamb	[129]
	*Solanum dulcamara* Linnaeus	[48]
	*Solanum lyratum* Thunb.	[131]
	*Solanum puberulum* Nuttal ex Seemann	[131]
	*Nicotiana longiflora* Cav.	[124]
	*Nicotiana tabacum* L.	[15]
	*Nicotiana rustica* L.	[48]
	*Nicotiana glauca* (Graham)	[82]
	*Datura stramonium* L.	[124]
	*Datura quercifolia* Kunth	[128]
	*Datura ferox* L.	[132]
	*Xanthium brasilicum* Vell.	[124]
	*Capsicum annum* L.	[41]
	*Capsicum frutescens* L.	[96]
	*Nicandra physalodes* (L.) Gaertner	[48]
	*Lycium halimifolium* Mill.	[48]
	*Lycium chilense* (Coralillo)	[131]
	*Lycium hirsutum* L.	[82]
	*Physalis peruviana* L.	[133]
	*Physalis angulata* L.	[130]
Amaranthaceae	*Amaranthus spinosus* L.	[124]
	*Amaranthus viridis* L.	[78]
	*Spinacia oleracea* L.	[126]
	*Beta vulgaris vulgaris* L.	[126,134]
	*Chenopodium bonus-henricus* (L.) Rchb.	[126]
	*Chenopodium rubrum* (L.) S. Fuentes, Uotila & Borsch	[126]
	*Chenopodium album* L.	[134]
Fabaceae	*Phaseolus vulgaris* L.	[135]
	*Medicago sativa* L.	[124]
	*Vicia faba* L.	[124]
Cucurbitaceae	*Citrullus lanatus* (Thunb.) Matsum. & Nakai	[124]
Convolvulaceae	*Convolvulus arvensis* L.	[134]
	*Calystegia sepium* (L.) Brown	[134]
Malvaceae	*Malva sylvestris* L.	[48]
Asteraceae	*Sonchus oleraceus* L.	[78]
	*Xanthium strumarium* L.	[136]
Poaceae	*Sorghum halepense* (L.) Pers.	[78]
Brassicaceae	*Sinapis arvensis* L.	[136]

**Table 2:** Insecticidal plants used to make botanicals used against *Tuta absoluta* (Eggs—E; Larvae—L; Pupa—P; Adults—A). The records were obtained from the literature at the time of writing and may not be purely exhaustive.

Natural Substance	Species	Host Developmental Stage	Reference
Botanicals	*Azadirachtin* spp.	E, L, P	[150]
	Petroleum ether extract	E, L	[150]
	*Jatropha curcus*	E, L	[150]
	*Allium sativum*	L	[156,157]
	*Ocimum basilicum*	L	[156,157]
	*Thymus vulgaris*	L	[156,157]
	*Ricinus communis*	L	[156,157]
	*Eucalyptus* spp.	L	[156,157]
	*Melia azedarach*	L	[156,157]
	*Geranium* spp.	L	[156,157]
	*Allium cepa*	L	[156,157]
	*Citrus aurantium*	L	[158]
	*Piper amalago* var. *medium*	L	[153]
	*Piper glabratum*	L	[153]
	*Piper mikanianum*	L	[153]
	*Simmondsia chinensis*	L	[151]

**Table 3:** Microbials and other natural substances used as biopesticides against *Tuta absoluta.* (Eggs—E; Larvae—L; Pupa—P; Adults—A). The records were obtained from the literature at the time of writing and may not be purely exhaustive.

Type of Microbial	Species	Host	Reference
	*Bacillus thuringiensis*	L	[160,169]
Entomopathogens	*Bacillus thuringiensis kurstaki*	L	[169]
	*Beauveria bassiana*	L	[169]
	*Metarhizium beauveria*	L	[170]
	*Metarhizium anisopliae*	L	[170]
	*Baculoviruses* (NPVs)	L	[171]
	*Saccharopolyspora spinosa*	L	[172]
Entomopathogenic nematodes	*Steinernema affine*	L	[172]
	*Steinernema carpocapsae*	L	[172]
	*Steinernema feltiae*	L	[62]
	*Heterorhabditis bacteriophora*	L	[62]
Other NSs	Pheromones	A	[36,59]
	Antimicrobial peptides (AMPs)	E, L, P	[173]

**Table 4:** Natural enemies (parasitoids and predators) reported in literature for *Tuta absoluta.* The list may not be purely exhaustive but was compiled using the available literature at the time of writing. (Eggs—E; Larvae—L; Pupa—P).

Natural Enemy	Species	Host	Reference
Parasitoids	*Agathis fuscipennis*	L	[131]
	*Apanteles dignus*	L, P	[15,16]
	*Apanteles gelechiidivoris*	L	[15,16]
	*Baryscapus bruchophagi*	L	[220]
	*Brachymeria secundaria*	L	[220]
	*Bracon lucileae*	L	[15,16]
	*Bracon lulensis*	L	[15,16]
	*Bracon* spp.	P	[15,16,119]
	*Bracon tutus*	L	[15,16]
	*Campoplex haywardi*	L	[15,16]
	*Capidosoma desantis*	E	[15,16]
	*Capidosoma koehleri*	E	[15,16]
	*Cheolras semele*	-	[221]
	*Closterocerus clarus*	L	[220]
	*Clostrocerus formosus*	L	[15,16]
	*Copidosoma desantisi*	E	[15,16]
	*Copidosoma koehleri*	E	[15,16]
	*Diadegma* spp., *D. ledicola* and *D. pulchripes*	P	[15,16,119]
	*Diglyphus crassinervis*	L	[221]
	*Diglyphus isaea*	L	[221]
	*Dineulophus phthormiaeae*	L	[15,16]
	*Dolichogenidea litae*	-	[221]
	*Elachertus inunctus*	L	[222]
	*Elasmus phthorimaeae*	L	[221]
	*Encarsia porteri*	E	[223]
	*Goniozus nigrifemur*	L	[15]
	*Habrobracon didemie*	L	[220]
	*Habrobracon hebetor*	L	[220]
	*Habrobracon nigricans*	L	[16,224]
	*Habrobracon osculator*	L	[119]
	*Halticoptera aenea*	L	[222]
	*Hemiptarsenus zilahisebessi*	L	[225]
	*Hockeria unicolor*	L	[220,221]
	*Horismenus sp*	P	[15,16,119]
	*Hyposoter didymator*	-	[226]
	*Necremnus artynes*	L	[218,225]
	*Necremnus metalarus*	L	[206]
	*Necremnus tidius*	L	[227]
	*Neochrysocharis formosa*	L	[119]
	*Neochrysocharis formosa*	L	[222,224]
	*Neochrysocharis formosa*	L	[15,16]
	*Pnigalio cristatus*	L	[220]
	*Pnigalio incompletus*	-	[220]
	*Pnigalio soemius*	L	[221]
	*Pnigalio* sp. *soemius complex*	L	[222]
	*Pseudapanteles dignus*	L	[15,16]
	*Pteromalus intermedius*	L	[220]
	*Pteromalus semotus*	-	[221]
	*Retisympiesis phthorimaea*	L	[15,16]
	*Retisympiesis phthorimaea*	L	[15,16]
	*Temelucha anatolica*	-	[221]
	*Trichogramma achaeae*	E	[214]
	*Trichogramma achaeae*	E	[224]
	*Trichogramma bactrae*	E	[15,16]
	*Trichogramma bourarachae*	E	[228]
	*Trichogramma dendrolimi*	E	[15,16]
	*Trichogramma exiguum*	E	[15,16]
	*Trichogramma fasciatum*	E	[15,16]
	*Trichogramma lopezandinensis*	E	[15,16]
	*Trichogramma minutum*	E	[15,16]
	*Trichogramma nerudai*	E	[15,16]
	*Trichogramma pintoi*	E	[15,16]
	*Trichogramma pretiosum*	E	[15,16,213]
	*Trichogramma rojasi*	E	[15,16]
	*Trichogramma telengai*	E	[15,16]
	*Zoophthorus macrops*	-	[221]
Predators	*Amblyseius cucumeris*	E, L	[229]
	*Amblyseius swirskii*	E, L	[229]
	*Brachygastra lecheguana*	L	[15]
	*Calosoma granulatum*	L, P	[15]
	*Coleomegilla maculata*	E, L	[15]
	*Cycloneda sanguinea*	E	[15]
	*Dicyphus errans*	E, L	[230]
	*Dicyphus maroccanus*	E, L	[230]
	*Dicyphus. tamaninii*	E, L	[206,229]
	*Doru lineare*	E	[15]
	*Engytatus varians*	E	[229]
	*Eriopsis conexa*	E	[15]
	*Franklinothrips vespiformis*	L	[15]
	*Labidura riparia*	P	[15]
	*Lebia concina*	L, P	[15]
	*Macrolophus pygmaeus*	E, L	[231]
	*Nabis ibericus*	L	[229,232]
	*Nesidiocoris tenuis*	E	[231]
	*Orius albidipennis*	-	[230,233]
	*Orius insidiosus*	E, L	[15]
	*Podisus nigrispinus*	L	[15]
	*Polistes carnifex*	L	[15]
	*Polistes melanosoma*	L	[15]
	*Polistes versicolor*	L	[15]
	*Polybia ignobilis*	L	[15]
	*Polybia scutellaris*	L	[15]
	*Protonectarina sylveirae*	L	[15]
	*Protopolybia exigua*	L	[15]
	*Scolothrips sexmaculatus*	L	[15]
	*Solenopsis geminata*	L, P	[15]
	*Solenopsis saevissima*	L, P	[15]
	*Synoeca cyanea*	L	[15]
